# Genome-wide expression analysis of *LACS* gene family implies *GhLACS25* functional responding to salt stress in cotton

**DOI:** 10.1186/s12870-024-05045-0

**Published:** 2024-05-13

**Authors:** Yuchen Xu, Shouyang Fu, Yiwen Huang, Dayun Zhou, Yuzhen Wu, Jun Peng, Meng Kuang

**Affiliations:** 1 Institute of Cotton Research of Chinese Academy of Agricultural Sciences/State Key Laboratory of Cotton Biology, Anyang, Henan 455000 China; 2 Sanya National Nanfan Research Institute, Chinese Academy of Agricultural Sciences, Sanya, Hainan 572024 China; 3 Henan University/State Key Laboratory of Crop Stress Adaptation and Improvement, Kaifeng, Henan 475004 China

**Keywords:** LACS, Abiotic stress, Yeast, VIGS, Cotton

## Abstract

**Background:**

Long-chain acyl-coenzyme A synthetase (LACS) is a type of acylating enzyme with AMP-binding, playing an important role in the growth, development, and stress response processes of plants.

**Results:**

The research team identified different numbers of *LACS* in four cotton species (*Gossypium hirsutum*, *Gossypium barbadense*, *Gossypium raimondii*, and *Gossypium arboreum*). By analyzing the structure and evolutionary characteristics of the *LACS*, the *GhLACS* were divided into six subgroups, and a chromosome distribution map of the family members was drawn, providing a basis for further research classification and positioning. Promoter cis-acting element analysis showed that most *GhLACS* contain plant hormones (GA, MeJA) or non-biological stress-related cis-elements. The expression patterns of *GhLACS* under salt stress treatment were analyzed, and the results showed that *GhLACS* may significantly participate in salt stress response through different mechanisms. The research team selected 12 *GhLACSs* responsive to salt stress for tissue expression analysis and found that these genes are expressed in different tissues.

**Conclusions:**

There is a certain diversity of *LACS* among different cotton species. Analysis of promoter cis-acting elements suggests that *GhLACS* may be involved in regulating plant growth, development and stress response processes. *GhLACS25* was selected for in-depth study, which confirmed its significant role in salt stress response through virus-induced gene silencing (VIGS) and induced expression in yeast cells.

**Supplementary Information:**

The online version contains supplementary material available at 10.1186/s12870-024-05045-0.

## Background

The Long-chain acyl-CoA synthetase superfamily, also known as acyl activating enzymes, comprises a significant type of enzyme called long-chain acyl-CoA synthetases (LACSs) [1, 2]. Most enzymes in this superfamily catalyze reactions through a two-step mechanism. The initial step involves the fusion of ATP with free fatty acids to form an adenylated intermediate. The subsequent step binds this intermediate with the thioester bond of CoA to produce acyl-CoA [2]. Generally, LACSs participate in the esterification process of long-chain free fatty acids (LCFAs), leading to the formation of fatty acyl-CoA thioesters. These products can subsequently enter various metabolic pathways, including but not limited to lipid storage, membrane lipids, surface lipids, and fatty acid degradation [3]. Additionally, some LACSs have been identified as transporters of fatty acids between different subcellular compartments, indicating their essential role in intracellular lipid metabolism [3, 4].

Fatty acids are substrates for LACS and are crucial for plant metabolism and resistance to abiotic and biological stresses. In plants, fatty acids serve both as an energy source and a protective surface layer against multiple stresses [5, 6]. *Arabidopsis thaliana* is a widely studied model plant, and its *LACS* family includes 9 members [7]. Numerous LACSs, confirmed to play a significant role in the production of lipids in the stratum corneum, are integral for plants as they minimize non-stomatal water loss. This crucial function enhances the plant’s resistance to stress. Several single or double mutants of *Arabidopsis thaliana LACS*, such as *atlacs2*, *atlacs1 atlacs2*, *atlacs4 atlacs8*, and *atlacs4 atlacs9* have been identified so far. These mutants demonstrated enhanced stratum corneum permeability, elevated water loss, and heightened susceptibility to drought [1, 8–10]. Due to functional redundancy, high-level *atlacs* mutants usually show higher sensitivity to drought. For example, the triple mutant *atlacs1 atlacs2 atlacs4* is a good example. It was found that the transpiration rate of triple mutant *atlacs1 atlacs2 atlacs4* exceeded that of single mutant or double mutant [4, 10]. In addition, in other species besides *Arabidopsis thaliana*, studies have described the role of *LACS* in stress resistance. Research discovered that ectopic expression of two *LACS* (*MdLACS2* and *MdLACS4*) in apple resulted in decreased epidermal permeability, reduced water loss, and increased resistance to drought and salt stress in transgenic plant [11]. Furthermore, another study demonstrated improved tolerance to PEG, NaCl, and ABA treatments in callus altered by *MdLACS1* [12]. The survival and thriving of plants in any given environment is attributed to the equilibrium between genetic adaptability and phenotypic plasticity, with abiotic environmental elements potentially triggering a stress response. These adaptive and responsive abilities can improve the reproductive adaptability of plants in the ecological environment and may be transformed into stable agricultural output. In summary, these results show that LACS activity is indispensable in plant growth, development, and stress resistance.

*Gossypium hirsutum* is a type of cultivated cotton which is well-known for its high fiber yield and has the characteristics of salt and alkali tolerance. It is the main pioneer crop in saline-alkali areas. However, external environmental factors and internal elements considerably limit the proliferation and productivity of cotton. Increasing research demonstrates that proteins from the *LACS* family have a significant function in the processes of seed germination, oil creation, and stress reaction. Nonetheless, there is relatively little research on LACS protein during cotton growth and development. This study comprehensively reviewed the *LACS* family of cotton and preliminarily identified the *LACS* of four major cotton species. In *Gossypium hirsutum*, we further studied one of the *LACS* and preliminarily identified its tolerance to saline-alkali environments. By analyzing the tolerance of some *LACS* gene to environmental stress, this study provides a new perspective for the evolution and in-depth study of this gene family. Our results provide an important basis for further revealing the mechanism of action of this family gene and the biological function of the *LACS* in growth and development.

## Results

### Identification of *LACS* family members

To identify the members of the *LACS* family in cotton, we used the hidden Markov model (HMM file) of PF05001 as the query condition and compared the proteome and genome of *Gossypium hirsutum* (ZJU), *Gossypium barbadense* (ZJU), *Gossypium arboreum* (CRI), and *Gossypium raimondii* (JGI) with local blast software. During this process, Based on the AMP-binding domain, we conducted screening and identified family members by excluding genes that do not contain this domain Finally, we identified 38, 39, 22, and 20 genes in *Gossypium hirsutum* (*GhLACSs*), Gossypium barbadense (*GbLACSs*), *Gossypium arboreum* (*GaLACSs*), and *Gossypium raimondii* (*GrLACSs*), respectively. According to the number of genes in four cotton species, it is determined that the *LACS* in cotton belongs to a conservative type, which is basically consistent with the evolution of allotetraploid cotton [13]. The genes were named *GhLACS1-38*, *GbLACSC1-39*, *GaLACS1-22*, *GrLACS1-20* according to their position on the chromosome.

Then we analyzed the physical and chemical properties of the LACS family members (Table S1), such as protein length, protein molecular weight (MW) and isoelectric point (PI). We found that in *Gossypium hirsutum*, the length of proteins encoded by LACS family members ranged from 535 aa (GhLACS13) to 762 aa (GhLACS36). The MW ranged from 59.29 kDa (GhLACS27) to 85.11 kDa (GhLACS36), and the PI ranged from 5.3 (GhLACS17) to 8.17 (GhLACS2). The encoded protein’s length and MW also fluctuate within a specific range in other cotton species. Furthermore, we predicted the subcellular location of these family members. The results showed that 25 genes in the *GhLACS* family were predicted to be located in plastids, 6 in the cytoplasmic matrix, 4 in the endoplasm, 2 in the nucleus, and 1 in the cell membrane.

### Analysis on the evolutionary relationship of family members

**Fig. 1:**
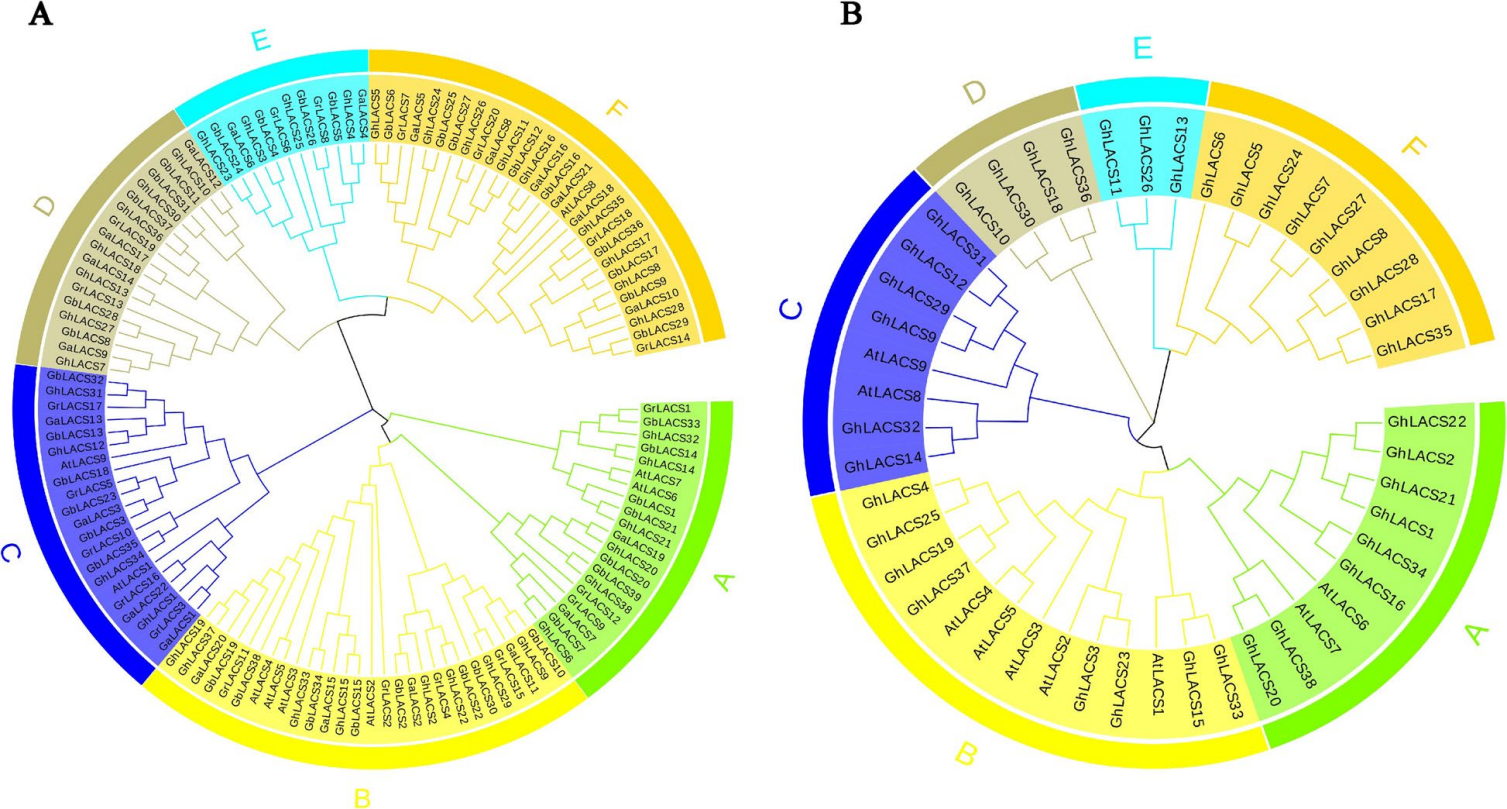
Phylogenetic tree of *LACS* family members. **A** The phylogenetic tree of four cotton species (*Gossypium hirsutum*, *Gossypium barbadense*, *Gossypium arboreum*, *Gossypium raimondii*) and *Arabidopsis thaliana LACS*; **B** The phylogenetic tree of *Gossypium hirsutum* and *Arabidopsis thaliana LACS*

In order to study the evolutionary relationship of the LACS protein family, you compared the full-length protein sequences of 128 protein sequences from *Arabidopsis thaliana* and four cotton species (Fig. S1). The evolutionary relationship between these sequences was constructed using MEGA7 software and a rootless phylogenetic tree was generated (Fig. 1A). In the results, LACS proteins were divided into six subgroups, and it was observed that subgroup F contained the most number of genes with 9 *GhLACSs*. It is worth noting that based on the number of genes contained in each subgroup in four cotton species, it was observed that the number of tetraploid cotton species *Gossypium hirsutum* is almost twice that of diploid cotton species, providing evidence that *Gossypium hirsutum* is the product of hybridization between two diploid cotton species (*Gossypium arboreum* and *Gossypium raimondii*). Furthermore, an analysis of the homologous genes of *Gossypium hirsutum* was conducted, and it was found that these genes evolved slowly after repeated events, which was conservative at the protein level. In the phylogenetic tree, all species have gene pairs from the same node, indicating that all species’ *LACS* experienced gene duplication events, which eventually led to the expansion of *LACS*. The replication of genes varied across different species and populations. Specifically, upland and island cotton both displayed a significantly higher gene count compared to other species, indicating a substantial evolutionary expansion of the *LACS* family in these two types of cotton. Additionally, a phylogenetic tree illustrating the evolutionary relationship between *Arabidopsis thaliana* and upland cotton was constructed (Fig. 1B), and it was found that there were no *AtLACS* in subgroup D, E, and F, while the *LACS* in subgroup D, E, and F might be unique to *Gossypium hirsutum*.

### Distribution of *LACS* family members on chromosomes

In order to further study the genetic differences of the *LACS* family members, we created the chromosome distribution map (Fig. S2-A) and statistical map (Fig. S2-B) of the *LACS* family members using the gff3 file and gene ID information from the genome. Through our research, we discovered that the *LACS* in *Gossypium hirsutum*, *Gossypium barbadense*, *Gossypium arboreum*, and *Gossypium raimondii* are located on specific chromosomes for each respective cotton variant. Furthermore, we found 118 family members on various specific chromosomes, with only the *GaLACS22* located on a scaffold. This result shows that the genetic evolution process of *LACS* is mature and stable. By examining the distribution position of genes, we discovered a gene in *Gossypium hirsutum* that is located in a similar position on chromosome 1 in both subgroup A and subgroup D. This discovery is similar to *Gossypium barbadense*. Where *GhLACS16* and *GhLACS34* are homologous genes in the evolutionary relationship and have similar promoter action elements. Notably, subgroup A of this cotton contains two additional genes compared to subgroup D. Furthermore, chromosomes A05 and D05 exhibit a higher gene count compared to other chromosomes in the genome.

The number of genes in *Gossypium barbadense* differs between the two subgroups. More genes are found on chromosomes 1 and 5 in both subgroup A and D compared to the other chromosomes. Interestingly, there is no gene distribution on chromosomes 4, 8, 12, and 13 in subgroup A and chromosomes 2, 8, 12, and 13 in subgroup D of the two tetraploid cotton species. This may be related to the chromosome deletion in these two cotton species during evolution. In *Gossypium arboreum*, one gene was mapped to a scaffold, and there are 4 genes on Chr01 and Chr05, while there is no gene on Chr08, and Chr13. However, in *Gossypium raimondii*, there are more genes on Chr02, and Chr09, with 4 genes on each chromosome, while Chr04, Chr08, and Chr13 of this cotton species have no *LACS* family gene distribution. The allocation of *LACS* across the 13 chromosomes among different cotton species exhibits an uneven distribution. Interestingly, there is no apparent correlation between the number of genes allocated to each chromosome and the respective chromosome length.

### Motif and gene structure analysis of conserved protein of GhLACS

**Fig. 2:**
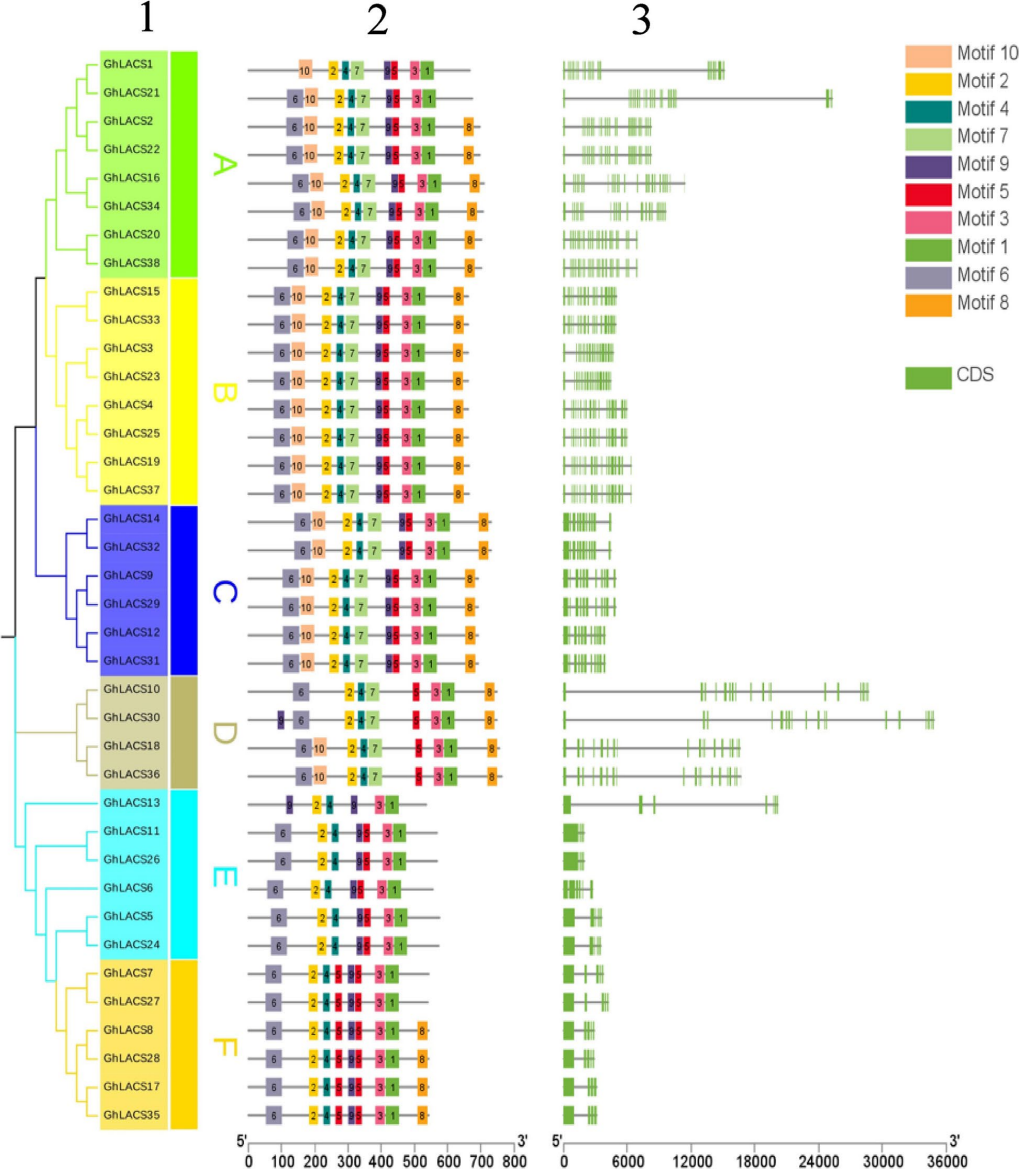
Phylogenetic tree (1), conserved domain (2) and gene structure (3) analysis of GhLACs

The MEME online website was utilized to examine the amino acid sequence encoded by the *GhLACS*, which revealed a total of 10 motifs (Fig. 2). These motifs perform different functions and are distributed in the sequences of various subgroups. In subgroup A, the length of the gene sequence varies significantly, including 8–10 motifs, and their gene structures also show considerable differences. However, both subgroup B and subgroup C have similar motifs, with 10 motifs and frequent intron intervals. Most members of subgroup D include motifs 6, 2, 4, 7, 5, 3, 1, and 8, and the intron length in this subgroup is generally long. Regarding the members of subgroup E, except for GhLACS13, they all contain motifs 6, 2, 4, 9, 5, 3, and 1. Interestingly, GhLACS13 contains two motif 9. With the exception of GhLACS7 and GhLACS27, most members of subgroup F contain 9 motifs. Similar motifs are found within the same subgroup, indicating that the protein structure is conserved within a specific subgroup. The functions of most conserved motifs still need to be clarified. Nevertheless, there is little difference in the exon length of the gene coding sequence, suggesting a certain level of conservation within the gene. Each subgroup exhibits high consistency in gene structure and phase distribution, further supporting the reliability of the homologous grouping relationship.

### Analysis of promoter cis-acting elements and expression

**Fig. 3:**
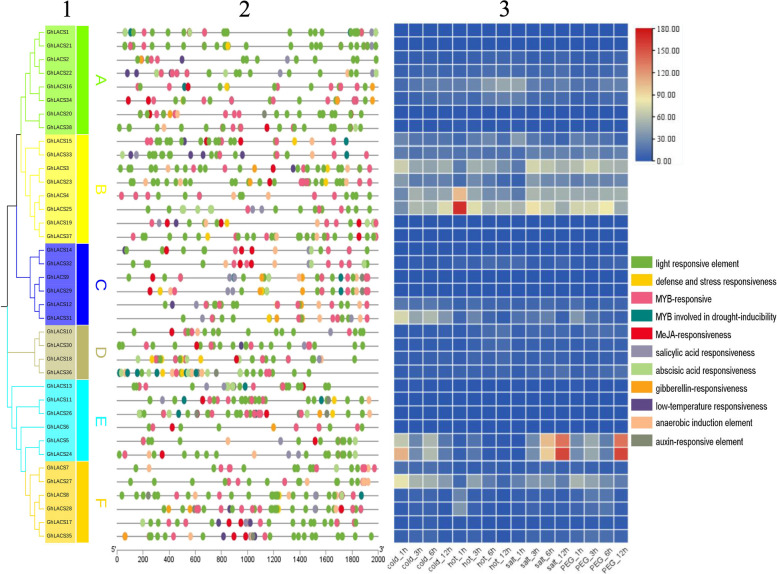
Analysis of *GhLACS* family promoters and differentially expressed genes. **1** Phylogenetic tree of *GhLACS*; **2** Cis-element in the promoter of *GhLACS* gene; **3** Expression profiles of members of the *GhLACS* gene family in *Gossypium hirsutum* under abiotic stress (cold, hot, salt, PEG)

To investigate the importance of various promoter elements in abiotic stress response, we examined the evolutionary relationship sequence of the *GhLACS* family along with the sequence of the 2000 bp region preceding the initiation codon of its members (Table S3). Our analysis identified the response elements of the *GhLACS* family to abiotic stresses such as plant hormones, drought, light, and low temperature (Fig. 3). Among them, the number of light response elements is the largest, followed by MYB response elements. Most members contain methyl jasmonate (MeJA) response elements and salicylic acid response elements, while about half of the members contain gibberellin response elements. Notably, the jasmonic acid pathway has been proven to be related to stress response [14, 15]. Furthermore, research has demonstrated the involvement of MeJA, a plant growth and development regulator, in the regulation of gene expression in plants [16–18]. Exogenous MeJA can promote the accumulation of osmotic substances such as proline and soluble sugar, which is beneficial to the osmotic adjustment of plants to adapt to waterlogging stress [19]. Additionally, under salt stress conditions, the use of MeJA can enhance the salt tolerance of crops. Enhancing crop salt tolerance not only mitigates the impact of salt stress on crop growth and development but also indirectly modifies the soil environment [20]. A large number of cis-acting elements are related to growth and development and plant hormone response, indicating that *GhLACS* may involve very complex regulatory patterns in the transcription process. Within the same gene subgroup, the homeopathic elements contained in gene promoters are different, even for homologous genes with high similarity. Through the analysis of promoters, we can acquire a more profound comprehension of how the gene family responds to diverse plant hormones, aiding in the comprehensive understanding of the regulatory network of the *GhLACS* family.

To understand the response mechanism of *GhLACS* to abiotic stress, we downloaded RNA-seq data (PRJNA248163) from the NCBI database for analyzing the expression patterns of these genes under various stresses (cold, hot, salt, and PEG). A total of 37 genes of FPKM were found in the RNA-seq data (Table S4), and heat maps were generated based on the expression levels of these genes under cold, hot, salt, and PEG stress (Fig. 3). The results indicated that nearly half of *GhLACS* showed no significant differential expression under various abiotic stress, while some genes were strongly induced by multiple stresses and exhibited clear differential expression, such as *GhLACS3*, *GhLACS5*, *GhLACS23*, *GhLACS24*, *GhLACS25*, *GhLACS31*, etc. Interestingly, gene expression within the same clade varied. Some genes were specifically induced by certain stress; for instance, *GhLACS31* exhibited high expression under cold stress but not under other stresses, while *GhLACS5* had minimal expression under heat stress but showed expression under salt stress. The number of significantly differentially expressed *GhLACS* genes under different stress conditions was calculated: 21 under cold treatment, 22 under high-temperature treatment, 17 under PEG treatment, and 22 under salt stress. The changes in gene expression levels under different stress conditions suggested that *GhLACS* members play a role in regulating abiotic stress.

### Collinearity analysis

We conducted a collinearity analysis on genes from four cotton species to determine their positional relationship, homology, amplification process, and arrangement sequence on the same chromosome. Firstly, we blasted the genome protein sequences of various cotton species. Then, we used MCScanX [21] to find the homologous gene pairs. Finally, combining the chromosome length files between genomes, we visualized the results using CirCos [22]. The results are presented in a circular diagram (Fig. S3). In total, 489 repetitive gene pairs were identified in the four cotton species (Table S5). Among them, 35 pairs were segmental duplications, and 454 pairs were whole-genome duplications. In upland cotton, out of the 38 genes, we found 40 gene pairs (Fig. S3-c), including 11 pairs of segmental duplications and 29 pairs of whole-genome duplications. In island cotton, out of the 39 *LACSs*, there were 42 gene pairs (Fig. S3-h), with 11 pairs of segmental duplications and 31 pairs of whole-genome duplications. Interestingly, there were no tandem duplications in the *LACS* of tetraploid cotton species. In *Gossypium arboreum*, 22 genes formed 8 pairs of *LACS* homologous genes (Fig. S3-d), all of which were segmental duplications. Similarly, *Gossypium raimondii* had four pairs of *LACS* homologous gene from 20 genes (Fig. S3-a), all of which were segmental duplications. These results indicate that segmental duplications/whole-genome duplication is the main reason for gene amplification, while tandem duplications does not seem to play a role in the evolution of the *LACS* family. In our analysis of diploids, we identified a total of 31 gene pairs in *Gossypium arboreum* to *Gossypium raimondii* (Fig. S3-f). In tetraploids, there were 108 gene pairs in *Gossypium hirsutum* to *Gossypium barbadense* (Fig. S3-e). There are 70 gene pairs in *Gossypium arboreum* to *Gossypium hirsutum* (Fig. S3-j), and 68 gene pairs linking *Gossypium arboreum* to *Gossypium barbadense* (Fig. S3-g). Furthermore, there are 58 gene pairs (Fig. S3-b) and 60 gene pairs (Fig. S3-i) linking *Gossypium raimondii* to *Gossypium hirsutum* and *Gossypium barbadense*, hkrespectively.

### Determination and analysis of selective pressure (Ka/Ks)

**Fig. 4:**
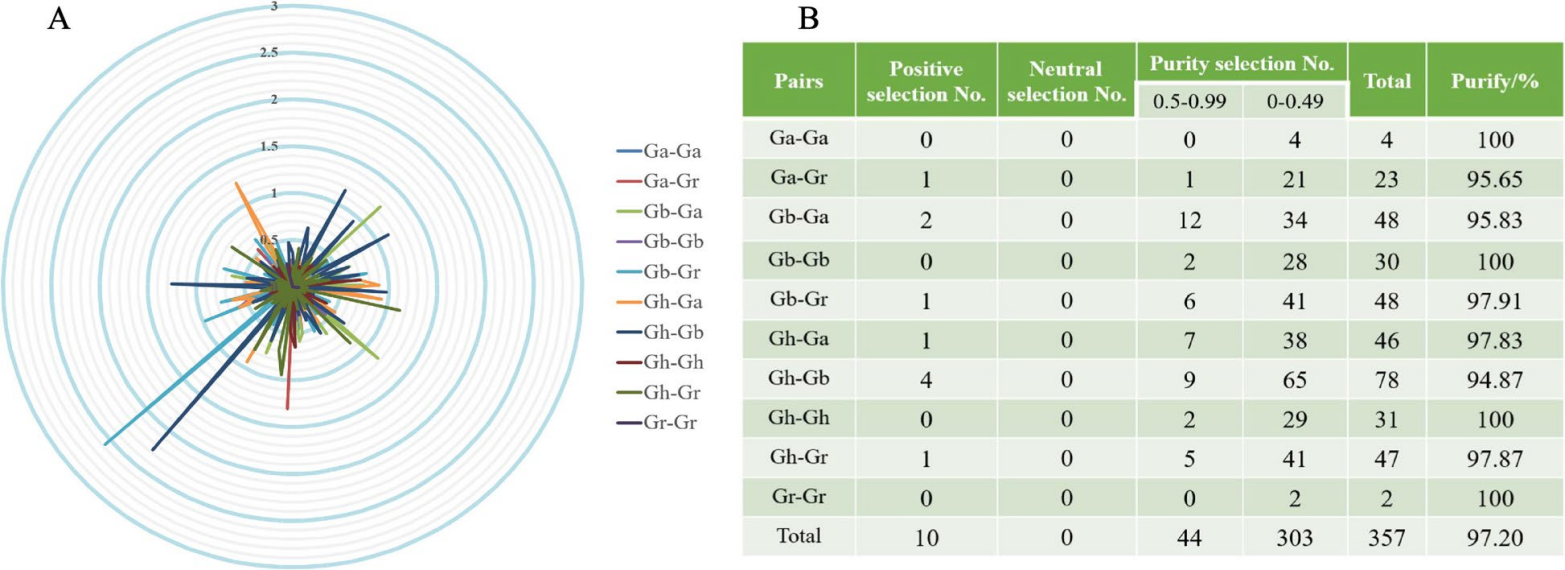
Selection pressure (Ka/Ks) analysis. **A** Select pressure (Ka/Ks) to analyze radar chart; **B** Prediction of no of duplicated gene pairs involved in different combinations from four *Gossypium* species

During the evolution of proteins, replicated genes may undergo three functional changes: non-functionalization, where they lose their original function; sub-functionalization, where they partially lose their former function; and new functionalization, where they acquire a new function [23]. We leverage the computation of the synonymous substitution rate (Ks) for synonymous (Ka) to deduce the level of selective constraint and additionally examine the selection pressure on gene pairs during the evolutionary process. We calculated the Ka, Ks, and Ka/Ks values for 357 pairs of homologous genes (Table S6) in ten combinations of four cotton species (Ga-Ga, Ga-Gr, Gb-Ga, Gb-Gb, Gb-Gr, Gh-Ga, Gh-Gb, Gh-Gh, Gh-Gr, Gr-Gr). A Ka/Ks ratio less than 1 is considered purification selection, indicating that natural selection eliminates harmful mutations and maintains protein integrity. A Ka/Ks ratio greater than 1 indicates positive selection, suggesting that natural selection plays a role in protein evolution. This causes the mutation location to become fixed rapidly in the population, subsequently accelerating gene evolution. On the other hand, a Ka/Ks ratio of 1 indicates neutral selection, implying that natural selection does not influence the mutation process [24]. By utilizing this Ka/Ks ratio, we assessed the selection pressure on recurring gene pairs. The findings revealed that Ka/Ks was greater than 1 in 10 gene pairs and less than 1 in 347 gene pairs. Furthermore, among the 303 gene pairs, the Ka/Ks values ranged from 0 to 0.49. Our research results show that the Ka/Ks ratio of approximately 97% of *LACS* pairs in four cotton species is less than 1 (Fig. 4). This suggests that the genes within this group are incredibly well-preserved, thoroughly filtered, and carefully selected, asserting their roles as crucial and difficult to alter.

### Specific expression of* LACS*

**Fig. 5:**
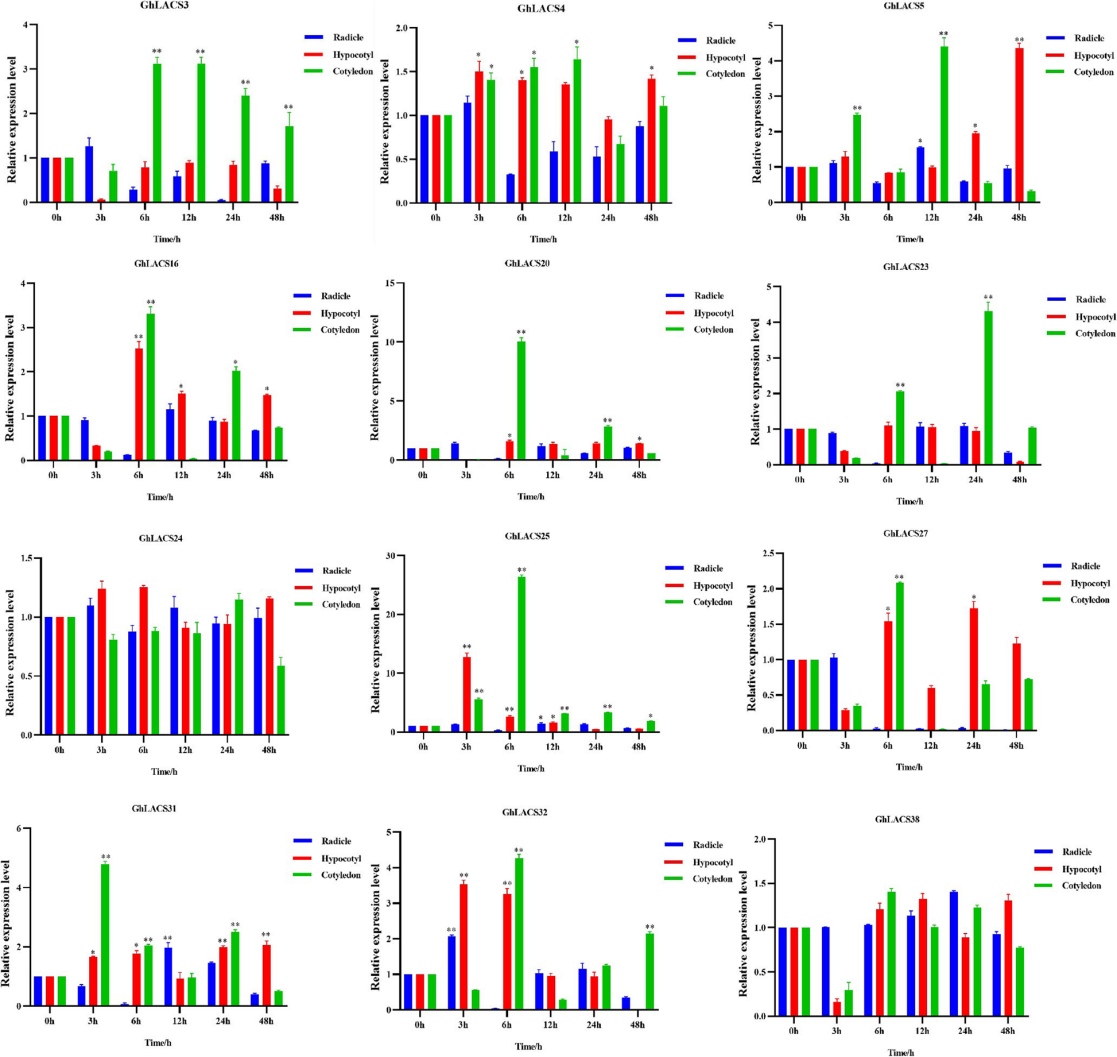
Expression level of *GhLACS* in different tissues. Asterisks indicate significant differences (*, *P*, 0.05; **, *P*, 0.01; Microsoft Excel Students’ t test.)

In order to gain a deeper understanding of the role of the *GhLACS* in plant growth and evolution, we initially selected 12 genes from different subgroups based on the phylogenetic tree. Subsequently, we employed qRT-PCR technology to analyze their expression patterns in various tissues, including roots, hypocotyls, and cotyledons. The results (Fig. 5) showed that most *GhLACS* members exhibited different degrees of response to salt stress, while *GhLACS24* and *GhLACS38* showed almost no response to salt stress in any of the examined parts. Specifically, *GhLACS23* only responded to salt stress in cotyledons, *GhLACS3* responded in both roots and cotyledons, while *GhLACS5*, *GhLACS16*, and *GhLACS20* responded in hypocotyls and cotyledons. Additionally, *GhLACS25*, *GhLACS31* and *GhLACS32* all demonstrated a certain degree of response. Notably, the *GhLACS25* exhibited a robust response to salt stress across all time intervals and in various tissue types. This response was particularly pronounced in both hypocotyls and cotyledons. The significant response of *GhLACS25* to salt stress may be linked to the presence of numerous anti-stress elements upstream of the *GhLACS*, which has further piqued our interest in this particular gene.

### VIGS of *GhLACS25*

**Fig. 6:**
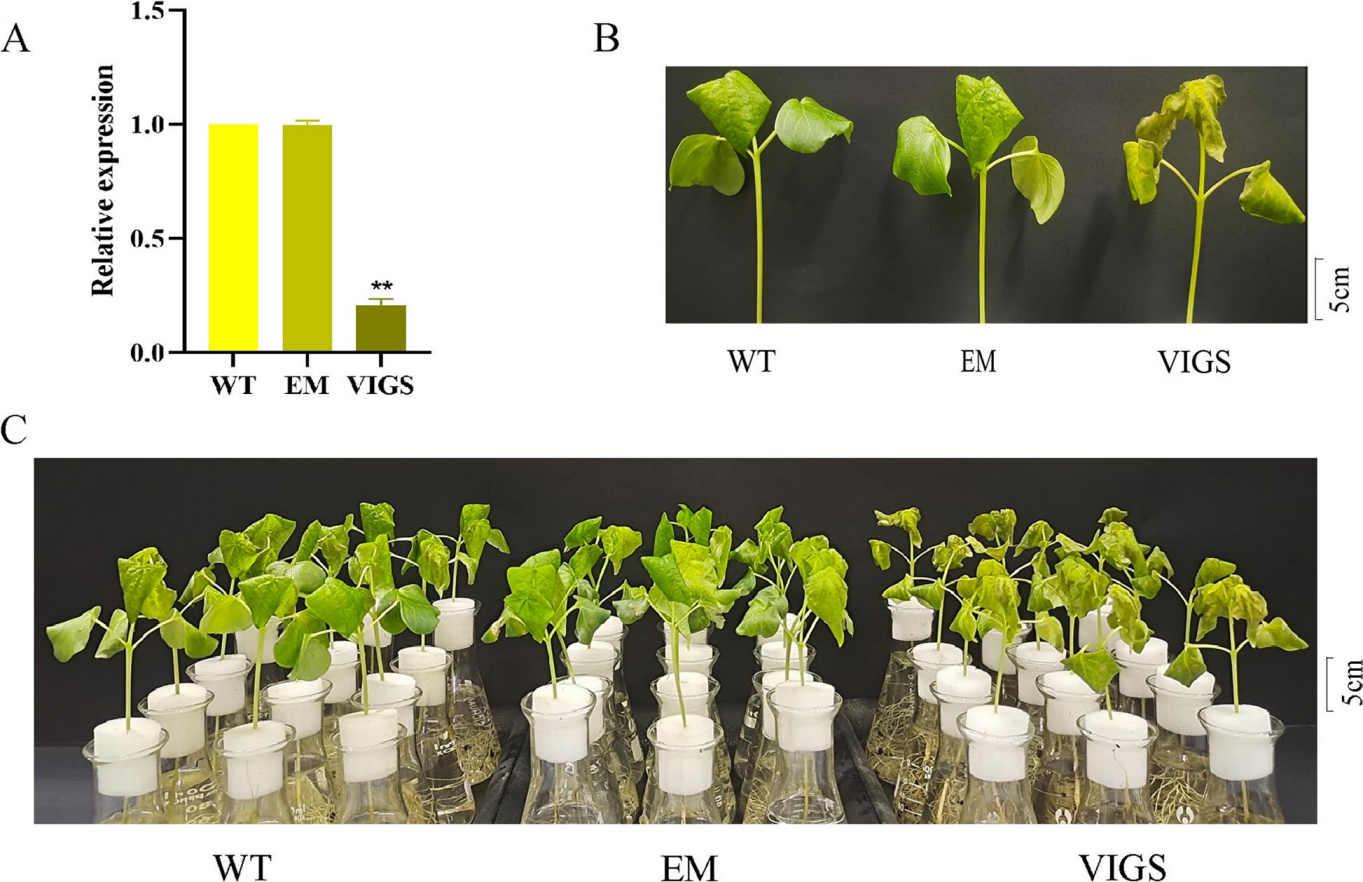
VIGS inquiry experiment. **A** Expression of *GhLACS25* in leaves infected by VIGS; **B** and **C** Phenotype of cotton infected by VIGS under salt stress for 2 days

Cotton cotyledons were infected with an empty vector (EM) and subjected to plant VIGS for 10–15 days, until the cotyledons were fully unfolded. Subsequently, we assessed the expression of the *GhLACS25* in wild-type (WT), EM, and VIGS plants using qRT-PCR (Fig. 6A). The results indicated that the expression level of the *GhLACS25* in the leaves of WT and EM plants was similar and significantly higher than that in VIGS plants, where the expression of the *GhLACS25* was barely detectable, confirming successful silencing of the *GhLACS2*5. Next, we treated WT, EM, and VIGS plants with 200 mM NaCl. After 2 days of treatment, it was observed that all three types of plants exhibited varying degrees of wilting. Infected WT and EM plants displayed more severe stress symptoms compared to uninfected WT plants, while VIGS plants showed the most pronounced stress symptoms, followed by EM plants and then WT plants (Fig. 6B and C). To some extent, these results indicate that the tolerance of *Gossypium hirsutum* plants to salt stress is affected after silencing the *GhLACS25*.

### Yeast induced expression

**Fig. 7:**
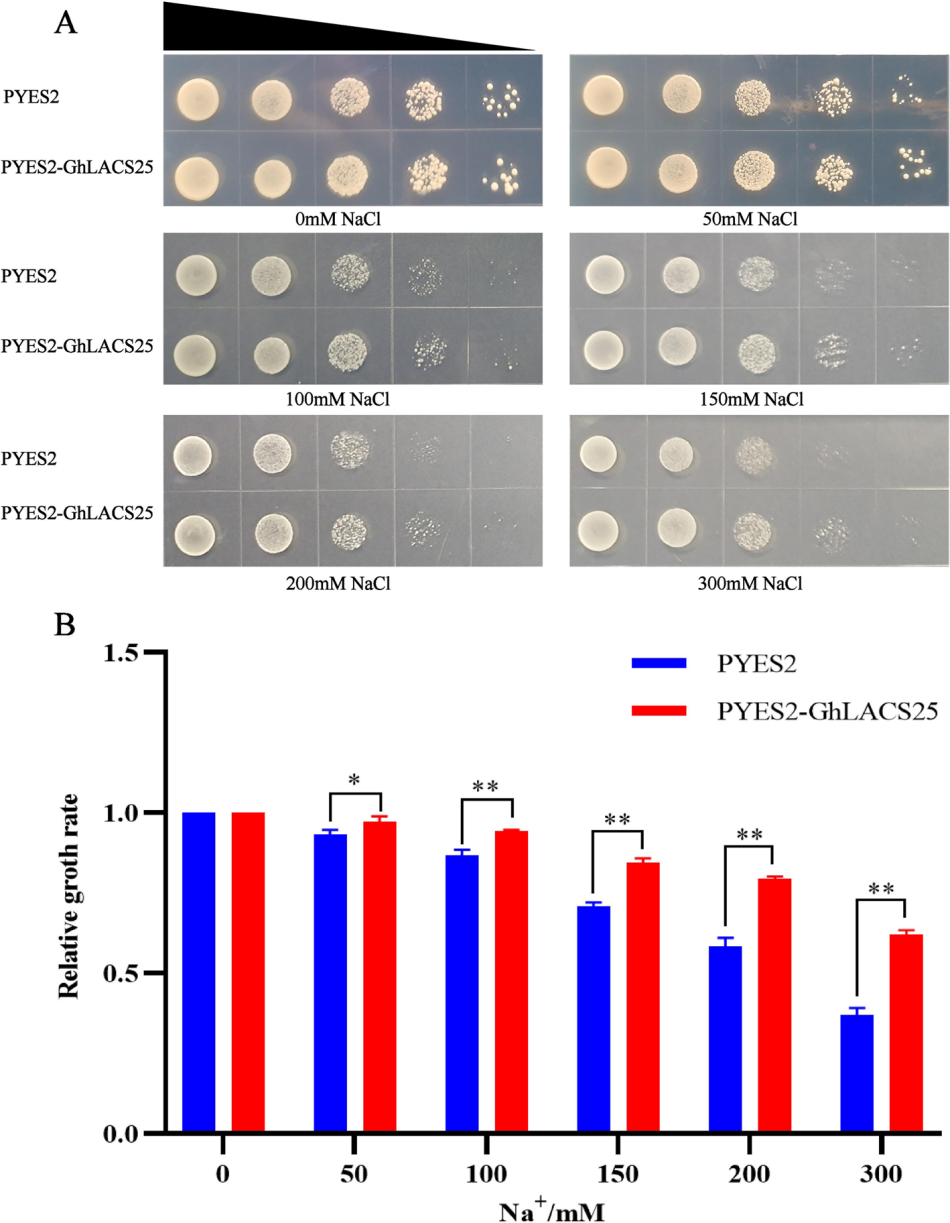
Functional analysis of PYES2-GhLACS25 in Na.^+^ sensitive strain INVSC1. **A** Growth status of expression vector PYES2-GhLACS25 and empty vector on SG-Ura with agar medium with different salt concentrations; **B** The relative growth rates of expression vector PYES2-GhLACS25 and empty vector on SG-Ura broth medium with different salt concentrations. The asterisk indicates the significant difference between PYES2 and PYES2-GhLACS25 (*, *P*, 0.05; **, *P*, 0.01; Microsoft Excel students’ t test)

Because the response to salt stress at the whole plant level is believed to largely depend on the tolerance mechanisms of cells [25]. *Saccharomyces* cerevisiae serves as a valuable model system for comprehending ion balance in plants due to the conservativeness of its basic transport mechanism [26]. In our study, we observed that PYES2-GhLACS25 and PYES2 were grown in SG-Ura agar with varying salt concentration gradients for 5 days. It was evident that the growth of the PYES2-GhLACS25 strain was weaker compared to that of PYES2 (Fig. 7A). Subsequently, we cultured PYES2-GhLACS25 and PYES2 in SG-Ura broth medium with different salt concentration gradients for 24 h, followed by measuring the OD value using a spectrophotometer. The relative growth rate of cells was determined based on the OD value (Fig. 7B). PYES2 and PYES-GhLACS25 exhibited different growth conditions under various salt concentrations. As the salt concentration increased, the growth inhibition of PYES2 yeast became more severe than that of PYES-GhLACS25 yeast, and this difference became increasingly apparent. By combining these two experimental results, we concluded that the expression of *GhLACS25* in yeast led to an increase in salt tolerance in yeast cells.

### Interaction network of GhLACS protein

**Fig. 8:**
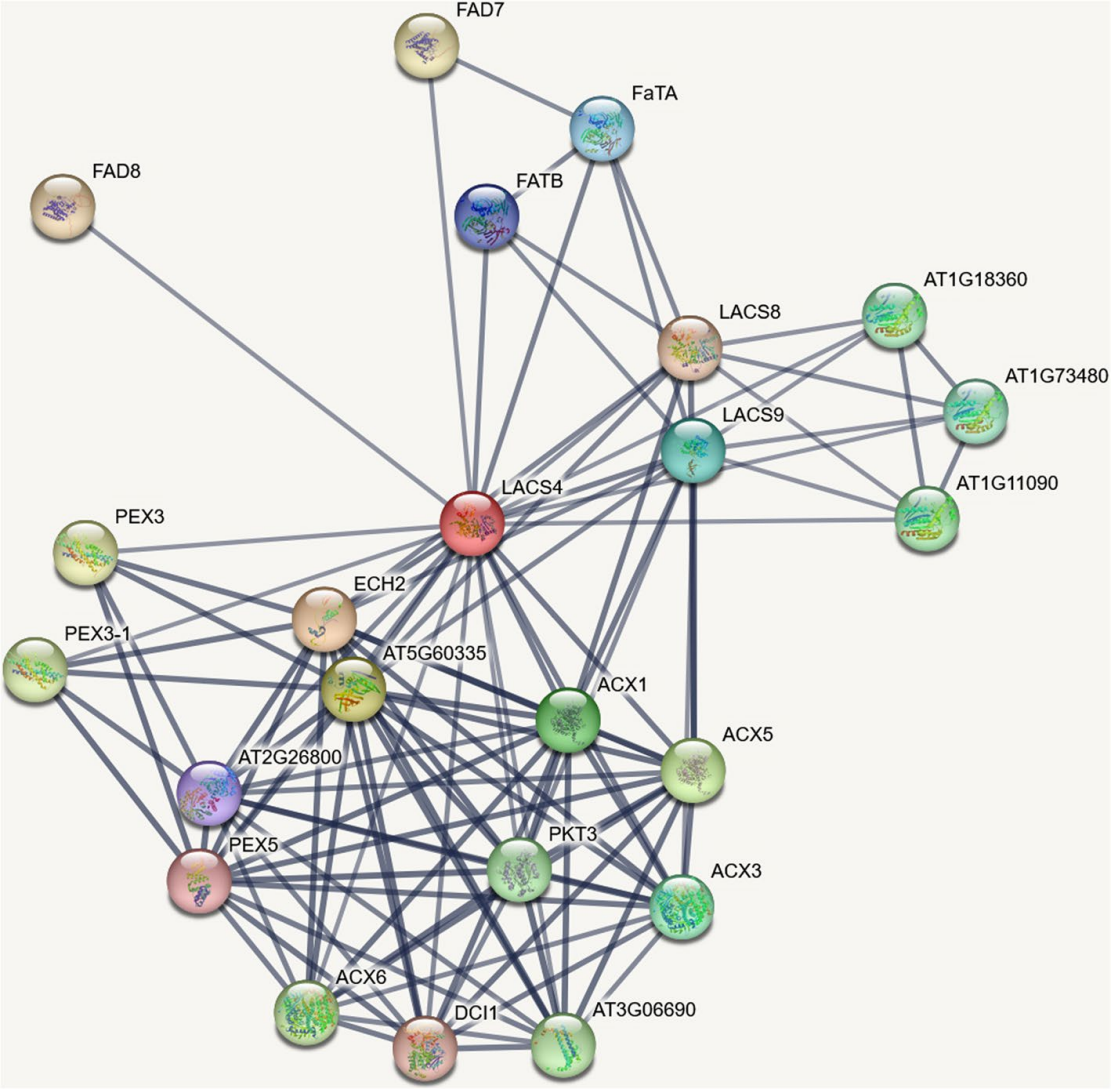
Interaction network of GhLACS protein

**Fig. 9:**
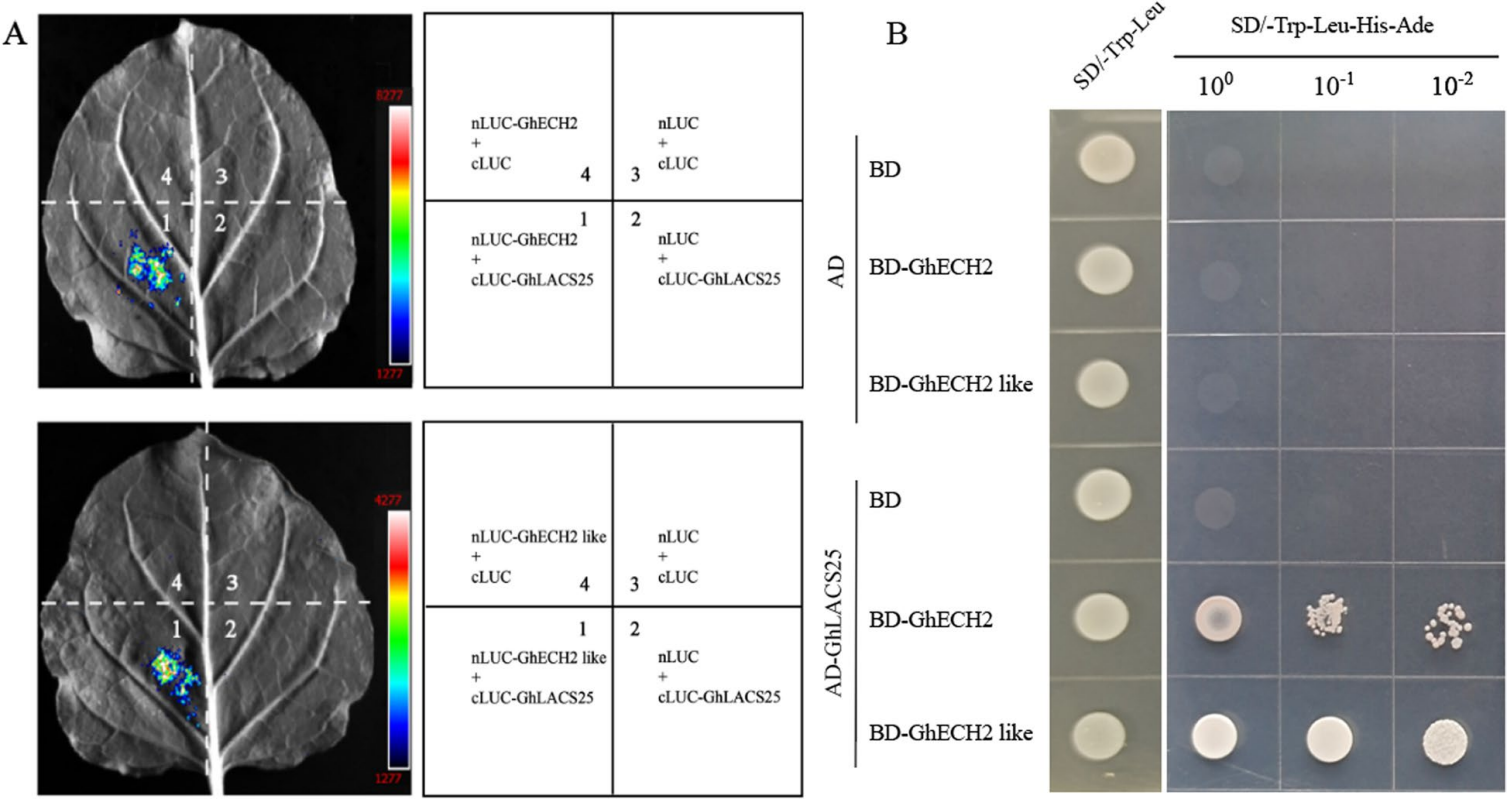
GhLACS25 interacts with GhECH2 and GhECH2 like. **A** Double fluorescein report detection experiment; **B** Yeast two-hybrid experiment

To gain insights into the function of the GhLACS protein, we used the STRING database to explore its interaction network and analyze the protein sequences of homologous genes in *Arabidopsis thaliana* (Fig. 8). By studying the AtLACS protein in *Arabidopsis thaliana*, we were able to infer the potential function of the GhLACS protein. Research findings indicate that the protein equivalent to GhLACS25 is AtLACS4. The fact that AtLACS4 interacts with AtLACS, a closely related member of the same family in *Arabidopsis thaliana*, suggests that GhLACS25 may also be involved in cellular lipid synthesis and subsequent breakdown through β-oxidation [27]. At5G60335 is a hydroxyl thioester dehydratase protein, which interacts with AtLACS4, suggesting that GhLACS25 may be involved in the dehydration process of 3-hydroxyl acp intermediate [28]. Additionally, the relationship between LACS4 and FATB could be crucial in providing saturated fatty acids necessary for plant growth and seed development [29]. The interaction between ECH2 and LACS4, which encode monofunctional alkenyl coenzyme a hydratase, indicates that GhLACS25 may be involved in the degradation of cis-unsaturated fatty acids [30]. In *Arabidopsis thaliana*, *ECH2* mutant leads to growth defects in seedlings [31], and ECH2 plays a role in ethylene signal transduction [32]. These findings underscore the significance of ethylene not only in the regulation of plant growth and development but also in how plants respond to various stresses [33, 34]. Interestingly, in cotton, the interaction between a GhECH2 protein and a GhECH2-like protein and GhLACS25 was verified by double-luciferase detection and yeast two-hybrid technique (Fig. 9), which further explained the important role of *LACS* in growth and development and resistance to stress.

## Discussion

The ever-evolving high-throughput sequencing technology has facilitated the sequencing of an increasing number of plant genomes. These scientific advancements greatly assist researchers in conducting genome-wide analyses of crucial gene families, as well as in identifying essential genes related to distinct characteristics or biological processes. Simultaneously, the results of these studies have accelerated the progress and realization of comprehensive plant genome sequencing projects. The number of *LACS* identified in *Gossypium hirsutum*, *Gossypium barbadense*, *Gossypium arboreum*, and *Gossypium raimondi*i is 38, 39, 22, and 20, respectively. The gene count in tetraploid cotton species, such as *Gossypium hirsutum* and *Gossypium barbadense*, is roughly equivalent to the combined gene count in two diploid cotton species like *Gossypium arboreum* and *Gossypium raimondii*. This observation partially elucidates the origin of tetraploid cotton species. Through the analysis of motif and gene structure, we infer that the *LACS* family in *Gossypium hirsutum* is relatively conservative, meaning that the gene sequence and structure are relatively stable. Additionally, the researchers also found that gene replication events may be caused by polyploidy, tandem, and segmental duplications [21]. In our chromosome mapping analysis, we found that *LACSs* were scattered on each chromosome in six subgroups. In our chromosome mapping analysis, we found that the genes of the 6 subgroups were scattered on each chromosome, and there was no *LACS* gene located on chromosome 8 of the diploid subgroup A, which was similar to the tetraploid subgroup A. Interestingly, there was distribution of *GrLACS20* gene on chromosome 12 of the diploid D subgroup, but in the two tetraploids, there were no genes on the chromosome 12 of the D subgroup.

Duplication events, including tandem duplication, segmental duplication, and whole-genome duplication, play an important role in gene amplification [35, 36]. The subfunctionalization model of repeated genes holds that after duplication events, each repeated gene retains only a part of the functions of its ancestors. This model can be further divided into complete subfunctionalization and partial subfunctionalization. Complete subfunctionalization means that two repetitive genes retain part of the functions of their ancestors without overlapping functions. In other words, each repetitive gene is responsible for different functions in cells or tissues. In this case, the loss of each gene will lead to the loss of function, so the existence of two genes is complementary. Partial subfunctionalization means that there is partial functional overlap between two repeated genes. Although they may have some common functions, they may also have specific functional differences in some aspects. In this case, the existence of two genes increases the functional diversity and provides a wider range of adaptation [37]. The subfunctionalization model of repetitive genes provides a mechanism to explain the functional changes and retention of repetitive genes during evolution. Through functional differentiation and subfunctionalization, repetitive genes can undertake more functions and provide greater adaptability to environmental changes and natural selection [38, 39]. This model provides an important theoretical basis for understanding the evolution of gene families and the formation of functional diversity [40]. The high proportion of repetitive genes in plant genomes reflects the retention rate of repetitive modules in plants [41]. It is still a subject of great interest to explore the retention mechanism of repeated genes in plant genomes. Whenever a duplication event occurs, the entire gene sequence of cotton is doubled, and these redundant genes are selectively recombined or lost over time [13]. Some repetitive genes may be preserved in future generations, thus providing the most basic genetic information for the evolution of plants [42]. Through the calculation of the Ks value of replicated genes, it is found that with the increase in the age of replicated genes, genes with fewer cis-acting elements are more common. The calculation of the Ka/Ks ratio revealed that approximately 97% of pairs of *LACS* in four cotton species had a Ka/Ks ratio less than 1, indicating that the gene family had been strongly purified. Together with the outcomes from the analysis of collinearity (Fig. S3), we identified 454 whole-genome duplications in four cotton species. Therefore, whole-genome duplication is the main reason for the amplification of *GhLACS*.

The *LACS* is a significant subset of the acyl-CoA synthase family, known for its ability to convert long-chain or ultra-long-chain fatty acids into their corresponding thioesters within plants. This enzyme plays a crucial role in numerous lipid-related metabolic processes. Changing in LACS activity can result in an array of phenotypic modifications, such as male sterility, fusion of organs, unusual epidermal structures, postponed seed germination, alterations in seed oil composition, and resilience to diverse environmental pressures such as drought, salt stress, hypoxia, and biological stress [43]. It is worth noting that cotton seeds have a high oil content [44], making it important to explore the relationship between the germination stage and stress resistance of cotton seeds. Several *atlacs* mutants have been found in *Arabidopsis thaliana*, exhibiting characteristics such as increased cuticle permeability, increased water loss, and drought sensitivity, suggesting their involvement in stress regulation and overlapping functions [8–10]. Our study found that the promoter region of the cotton *GhLACS* contain rich response elements to light, low temperature, drought, and various hormones (such as MeJA, SA, ABA, IAA, and GA). Furthermore, numerous elements correlating with hormone response have been identified within the promoter region of the *GhLACS*. Hormones like ABA, MeJA, and GA play pivotal roles in managing plant responses to various stress situations [45]. The presence of these elements aligns with the role of the *GhLACS* in augmenting cotton’s resistance to saline stress conditions, as demonstrated in *GhLACS25*. We speculate that *LACS* family may be involved in abiotic stress response and hormone regulation pathways in plants. Similar to *AtLACS1* and *AtLACS2* in *Arabidopsis thaliana*, *LACS* could potentially play a role in the plant’s response to abiotic stress, with concurrent roles in keratin and wax synthesis, providing protection to plants against different environmental pressures [1].

In plants, the LACS protein mainly exists in a few subcellular structures such as the endoplasmic reticulum, plastids, peroxisomes, and cytoplasm. Plastids are the main sites for fatty acid synthesis. *LACS* is widely distributed in eukaryotes, and its activity can be detected in subcellular structures such as plastids, microsomes, mitochondria, and peroxisomes [46, 47]. Several *LACS* exhibit varying expression patterns in diverse plant tissues and perform various functions. In this study, we examined 12 *GhLACSs*. *GhLACS24* and *GhLACS31* showed high levels of expression in the roots, while *GhLACS11*, *GhLACS24*, and *GhLACS26* were predominantly expressed in the leaves. *GhLACS25* was mainly expressed in both stems and leaves. Notably, *PoLACS4* in peony has been shown to rapidly respond to drought and salt stress, potentially activating a series of subsequent adaptation and resistance reactions [48]. The study of *MdLACS2* in apple demonstrated its ability to reduce epidermal permeability and water loss, thereby promoting drought resistance in transgenic plants [49]. Ectopic expression of *MdLACS4* can promote the accumulation of cuticle wax and enhance plant resistance to abiotic stress [11]. *GmLACS2-3* also plays an important role in preventing water loss [50]. Based on the expression profile analysis, we selected the *GhLACS25* gene, which exhibited positive responsiveness to salt stress and high expression levels in roots and leaves. We employed VIGS technology to silence the gene in cotton, which also led to its expression in yeast. The results of both experiments were consistent, indicating that *GhLACS25* plays a role in salt stress resistance. Additionally, we verified the interaction between the GhLACS protein and the GhECH2 protein, which is associated with plant growth and development [31]. These conclusive findings suggest a potential involvement of the *GhLACS* in the regulatory processes of cotton growth, development, and salt stress.

## Conclusion

In the above research, we identified the *LACS* family of four cotton species and analyzed their evolutionary relationships. By comparing the gene structure, chromosome distribution, phylogenetic relationship, cis-acting elements, and collinearity of the *GhLACS* in *Gossypium hirsutum,* we have gained a deeper understanding of the cotton *LACS* family. Additionally, by analyzing the cis-acting elements of the *GhLACS* promoter, we speculate that the *GhLACS* may be involved in stress defense response, abiotic stress, and plant hormone signal transduction mechanisms. Based on this, we selected 12 *GhLACS*s for simple tissue specificity analysis, using real-time quantitative data from the previous period. We then conducted VIGS experiments and yeast-induced expression experiments on *GhLACS* that showed significant responses under salt stress. Finally, we determined that the *GhLACS25* gene may be involved in the upland cotton’s response to salt stress, providing a strong basis for studying the molecular mechanism of the *LACS* in plants under abiotic stress.

## Materials and methods

### Plant materials and growth conditions

Cotton seeds were cultured in a greenhouse at 28 ℃ (16 h light and 8 h dark) from the Han M263 material preserved by the Cotton Research Institute of the Chinese Academy of Agricultural Science. The tobacco species was Benji tobacco, which was cultured in a greenhouse at 28 ℃ (16 h light for 8 h dark).

### Identification of members of upland cotton gene family

First, the cotton genome data were downloaded from the Cotton FGD website (http://www.cottonfgd.org/), including the genome data file, CDS sequence file, protein data file, and gff3 file. Next, the HMM file of the AMP-binding model was obtained from the PFAM website (https://pfam.xfam.org/) to identify LACS members in cotton. On a Windows system, you can run the HMMER 3.0 program and use the AMP-binding model for the hmmsearch program to compare and screen the genome data of the four cotton species. To obtain the initial members of the *LACS* family, a local blast was carried out using 9 LACS sequences from *Arabidopsis thaliana*. Sequences containing C, N, and CN residues were removed using the CD search on NCBI (https://www.ncbi.nlm.nih.gov/structure/CDD/wrpsb.cgi). Finally, the members of the *LACS* family from the four cotton species were obtained. We used SubPlant (http://bioinfo.usu.edu/Plant-mSubP/) to predict the subcellular localization information of *GhLACS*. Additionally, we utilized the Cotton FGD to search for other characteristics of the *LACS*. Furthermore, we analyzed information such as PI and MW using the online tool ExPasy-ProtParam (https://web.expasy.org/protparam/). These methods provide various data and information for research, which are helpful in fully understanding the characteristics and functions of the *GhLACS* and its encoded protein. This information will play an important supporting role in our research.

### Evolutionary analysis of gene system

The target sequence file is submitted to MEGA7 in fasta format, ClustalW algorithm was used to perform multiple sequence alignment and output meg format file. And the relationship between sequences is analyzed by neighborhood connection method [51]. By analyzing 1000 random data sets, Bootstrap analysis is used to evaluate the tree topology of neighbor connection data [52]. The phylogenetic tree is constructed with MEGA7, and the generated Newick file is beautified with the online website EvolView (https://www.evolgenius.info/evolView-v2/).

### Chromosome localization

The TBtools software was utilized to map the chromosome position of family members using the *Gossypium hirsutum* (ZJU) reference genome gff3 file and gene ID file. Similarly, the position of the *LACS* in the chromosomes of *Gossypium barbadens*e (ZJU), *Gossypium arboreum* (CRI), and *Gossypium raimondii* (JGI) was successfully mapped using the same method. To obtain the genome sequences of *Gossypium barbadense*, *Gossypium arboreum*, and *Gossypium raimondii*, we downloaded them from the Cotton FGD.

### Protein conserved domain and gene structure analysis

By analyzing the *LACS* family of *Gossypium hirsutum*, the conserved sequence of the protein was successfully identified using the MEME website (http://meme-suite.org/). For this analysis, a maximum motif parameter of 10 was used while keeping the other parameters unchanged, resulting in the generation of the *LACS* family domain file. To visualize the protein domain, the Domain MAST file, gene evolution relationship file, and *Gossypium hirsutum* genome gff3 file downloaded from the MEME website were employed in conjunction with TBtools [53].

### Collinearity analysis of *LACS* in four cotton species

The gff3 file of the target genome was prepared and integrated with TBtools software to generate the protein sequence alignment of the genome. Subsequently, gene pair files for each combination of two species (Ga-Ga, Ga-Gh, Ga-Gr, Ga-Gb, Gb-Gh, Gb-Gr, Gb-Gb, Gr-Gh, Gr-Gr, Gh-Gh) were obtained. The TBtools software was employed to analyze the collinearity relationship of repetitive gene pairs among the four cotton species. Finally, the collinearity results were visualized using the chromosome length file and genome comparison file.

### Analysis of *GhLACSs* promoter region

Firstly, we utilized the TBtools software to extract the upstream 2000 bp DNA sequence of the *GhLACS* from the genome sequence fa file. Next, we identified and predicted the cis-regulatory elements present in the promoter region of the *GhLACS* using the Plant CARE website (http://bioinformatics.psb.ugent.be/webtools/plantcare/html/). During this process, we focused on screening and analyzing cis-acting elements that were associated with abiotic stress, plant hormone metabolism, and plant growth and development. Finally, the relevant charts were redrawn using the TBtools software [53].

### Specific expression of* LACS*

The salt-tolerant cotton material (Han M263) seeds were generously provided by the State Key Laboratory of Cotton Biology. The cotton material was treated with water as the control and saltwater of 200 mM as the experimental group. To extract total RNA, we followed the instructions of the RNA EASY spin Plus plant RNA rapid extraction kit from Beijing Tiangen Biotechnology. The three tissues, namely young roots, hypocotyls, and cotyledons, were used for RNA extraction. Next, cDNA synthesis was performed using the Transscript all-in-one first-strand cDNA synthesis supermix for qPCR, following the provided guidelines for one-step gDNA removal. For the qRT-PCR experiment, we utilized the Roche 480II equipment and TransStart Top Green qPCR SuperMix fluorescence quantitative kit. The processing results were calculated using the ΔΔCt method [54] to calculate the processing results, and choose TBtools software for data analysis.

### VIGS in upland cotton

The linear pYL156 vector was obtained by using *Bam*HI and *Sac*I restriction sites, and the cloned vector T-GhLACS25 was used as a template to amplify with primer GhLACS25-V to obtain the VIGS fragment. In-FuSion technology was used to insert the VIGS silent fragment of *GhLACS25* into the linear pYL156 vector. First, the plasmid pYL156-GhLACS25 was transformed into *Agrobacterium* LBA4404. Then, recombinant *Agrobacterium* was obtained using a chemical method. When two cotyledons of upland cotton seedlings were fully expanded but no true leaves had grown, LB liquid medium containing 50 μg/ml kanamycin and 25 μg/ml rifampicin with *Agrobacterium* pYL156 (empty carrier) and pYL156-GhLACS25, and pYL192 as auxiliary bacteria, were used for culture. The mixture was incubated overnight in the dark on a shaker at 28 °C and 200 rpm. Once shaken to an OD_600_ = 1.2–1.5, a high-speed centrifuge was used to collect the bacteria (10 m at 5000 rpm). The supernatant was discarded, and an equal amount of aseptic suspensions (10 mM MES, 200 mM AS, 10 mM MgCl_2_, pH 5.8) was added and shaken well. The bacteria were allowed to resuscitate at room temperature in the dark for 4 h. Prior to infection, recombinant Agrobacterium, positive control, and blank control were re-suspended with an equal volume of adjuvant bacteria. A 1 ml sterile syringe was used to inject the mixture into the cuticle of the upland cotton cotyledon so that the mixture filled the entire cotyledon. After injection, the cotton seedlings were cultured in the dark for 24 h and then cultured normally. Two weeks later, the plants of pYL156 and pYL156-GhLACS25 were treated with 200 mM NaCl and their responses were observed. Finally, gene expression was analyzed through a fluorescence quantitative experiment.

### Construction of *GhLACS25* expression plasmid for yeast

The coding region of *GhLACS25* was cloned into vector PYES2(PYES2-GhLACS25) and then PYES2 (PYES2-GhLACS25) was transferred into INVSC1 chemically active cells (Weidi, YC1050). The yeast culture temperature was 30 ℃. Next, screening by SD-Ura solid medium showed that the positive bacteria. We let PYES2-GhLACS25 and PYES2 grow on SG-Ura with agar medium with different salt concentration gradients, and took photos five days later. Secondly, we put PYES2-GhLACS25 and PYES2 on SG-Ura broth medium with different salt concentration gradients. The rotating speed of the shaker is 250 rpm. After 24 h of culture, the absorbance was measured at the wavelength of 600 nm to calculate the relative growth rate of yeast.

### Luciferase complementation assays

Using the primer (Table S2) provided in the attached table, we cloned the coding region of *GhLACS25* into pCAMBIA1300-cLUC, and constructed the GhLACS25-cLUC vector. At the same time, the coding regions of *GhECH2* and *GhECH2-like* were cloned into pCAMBIA1300-nLUC to construct GhECH2-nLUC and GhECH2-like-nLUC vectors. Following a 3–4 weeks cultivation period, tobacco (Nicotiana tabacum) plants were prepared. An appropriate amount of Agrobacterium containing the target gene and the interacting gene vector was introduced into resistant LB medium (containing Kanamycin at 50 mg/L and Rifampicin at 25 mg/L) for culture until the OD_600_ reached approximately 0.8–1.0. Subsequently, the two types of bacterial liquid were mixed in equal proportions. After collecting and resuspending the bacterial liquid through centrifugation, it was injected into tobacco leaves. Prior to applying the substrate near the injection site, the infected plants were cultured in darkness for 24 h, followed by normal cultivation for 2 days. Finally, fluorescence was observed using a luminescence imaging system.

### Yeast two-hybrid assays

The coding region of *GhLACS25* was successfully cloned into the pGADT7 vector (AD-GhLACS25), while the coding regions of *GhECH2* and *GhECH2-like* were separately inserted into the pGBKT7 vector (BD-GhECH2 and BD-GhECH2-like, respectively). Subsequently, the constructs pGADT7 (AD-GhLACS25), pGbKT7 (BD-GhECH2), and pGBKT7 (BD-GhECH2-like) were introduced into Y2H Gold chemically active cells (Weidi, YC1002). To identify positive bacteria, SD-Trp-Leu (DDO) medium was used for screening, followed by transferring the colonies to SD-Trp-Leu-His-Ade (QDO) medium. After inoculation, the cultures were allowed to grow for 3 days, and photographs were taken to observe the results.

### Supplementary Information

**Additional file 1: Figure S1.** 128 protein sequences from *Arabidopsis thaliana* and four cotton species. **Figure S2.** Localization and quantitative statistics of *LACS* on chromosome. **Figure S3.** Co-linearity of *LACS* within and between genomes of four cotton species. **Table S1.** Attached table of physical and chemical properties. **Table S2.** Primer sequence. **Table S3.** Statistics of promoter cis-element. **Table S4.** RNA-seq data. **Table S5.** Tandem repeats and fragment repeats in four cotton species. **Table S6.** Gene pairs of ten combinatorial.

## Data Availability

The genomic data of cotton, Arabidopsis thaliana in the article can be downloaded from Cotton FGD (https://cottonfgd.org/) and TAIR (https://www.arabidopsis.org/) respectively. The analysis software, analysis methods and datasets generated are available from the corresponding author on reasonable request.

## Abbreviations

LACS: Long-chain acyl-coenzyme A synthetase
ECH2: Enoyl coenzyme hydratase 2
IAA: Auxin
VIGS: Virus induced gene silencing
EM: Empty vector
WT: Wild type
*At*: *Arabidopsis thaliana*
*Gh*: *Gossypium hirsutum*
*Gb*: *Gossypium barbadense*
*Ga*: *Gossypium arboreum*
*Gr*: *Gossypium raimondii*
MEME: Multiple Em for Motif Elicitation
MeJA: Methyl Jasmonate
