# Supplementary figures and images for: Genome-wide expression analysis of LACS gene family implies GhLACS25 functional responding to salt stress in cotton
Source: BMC Plant Biol. 2024 May 13;24:392. doi: 10.1186/s12870-024-05045-0 (PMC11089787; doi:10.1186/s12870-024-05045-0)

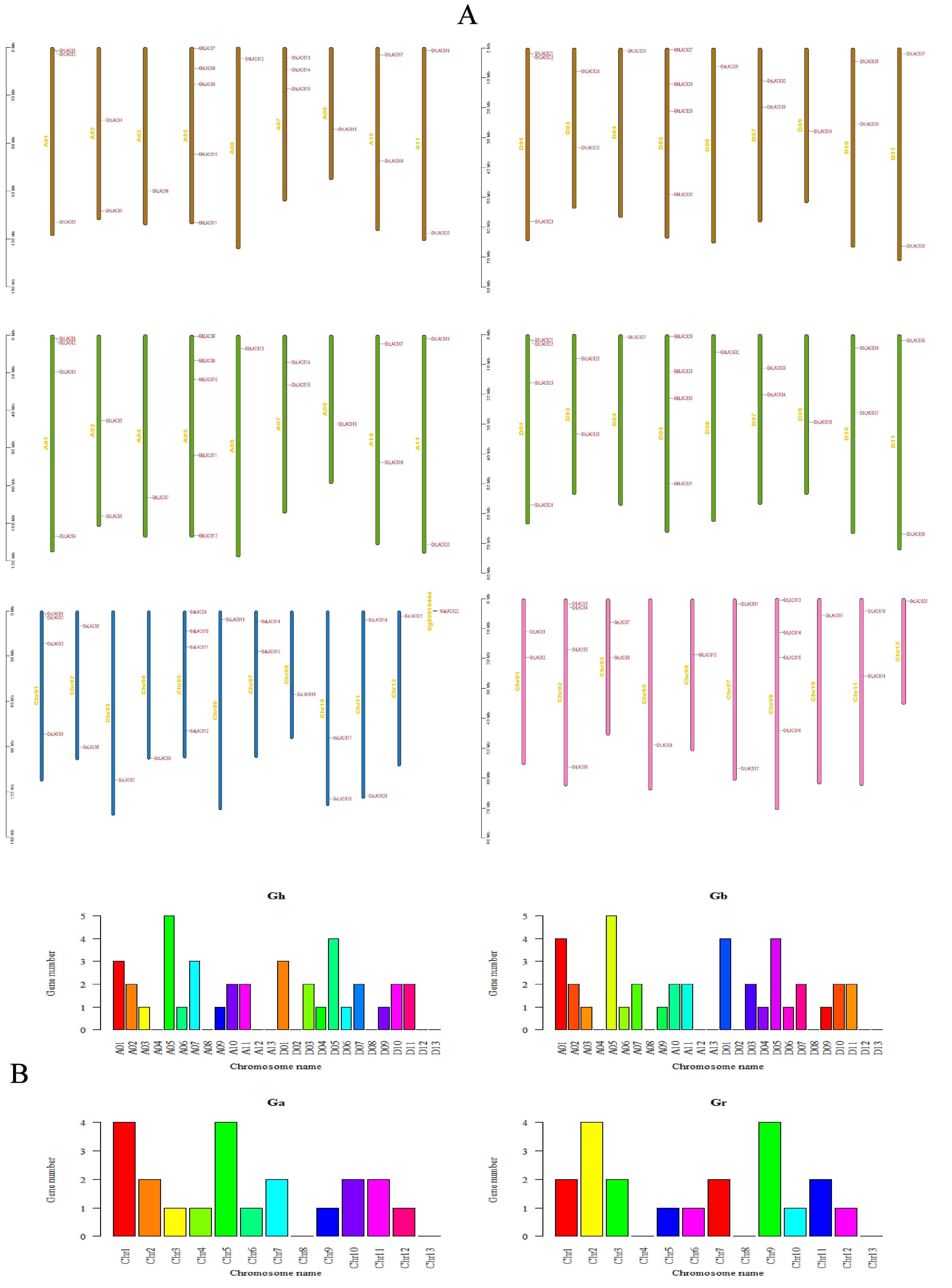

Supplement: Supplementary file 1 — Additional file 1: Figure S1. 128 protein sequences from Arabidopsis thaliana and four cotton species. Figure S2. Localization and quantitative statistics of LACS on chromosome. Figure S3. Co-linearity of LACS within and between genomes of four cotton species. Table S1. Attached table of physical and chemical properties. Table S2. Primer sequence. Table S3. Statistics of promoter cis-element. Table S4. RNA-seq data. Table S5. Tandem repeats and fragment repeats in four cotton species. Table S6. Gene pairs of ten combinatorial. [file 12870_2024_5045_MOESM1_ESM.zip › Supplementary Figure 2.jpg]

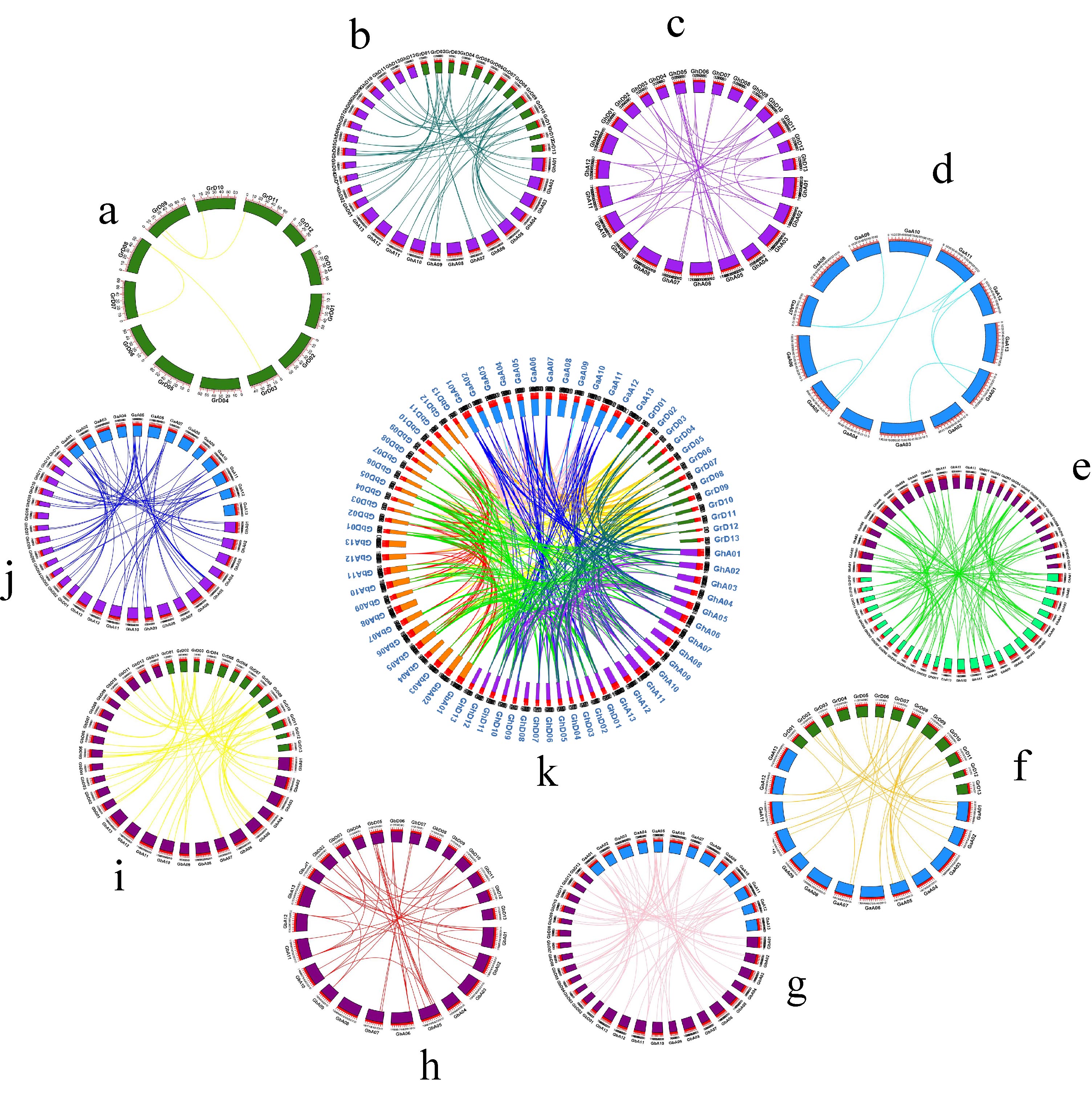

Supplement: Supplementary file 1 — Additional file 1: Figure S1. 128 protein sequences from Arabidopsis thaliana and four cotton species. Figure S2. Localization and quantitative statistics of LACS on chromosome. Figure S3. Co-linearity of LACS within and between genomes of four cotton species. Table S1. Attached table of physical and chemical properties. Table S2. Primer sequence. Table S3. Statistics of promoter cis-element. Table S4. RNA-seq data. Table S5. Tandem repeats and fragment repeats in four cotton species. Table S6. Gene pairs of ten combinatorial. [file 12870_2024_5045_MOESM1_ESM.zip › Supplementary Figure 3.jpg]
